# The Impact of Incretin-Based Medications on Lipid Metabolism

**DOI:** 10.1155/2021/1815178

**Published:** 2021-12-29

**Authors:** Habib Yaribeygi, Mina Maleki, Alexandra E. Butler, Tannaz Jamialahmadi, Amirhossein Sahebkar

**Affiliations:** ^1^Research Center of Physiology, Semnan University of Medical Sciences, Semnan, Iran; ^2^Urology and Nephrology Research Center, Shahid Beheshti University of Medical Sciences, Tehran, Iran; ^3^Research Department, Royal College of Surgeons in Ireland, PO Box 15503, Adliya, Bahrain; ^4^Department of Food Science and Technology, Quchan Branch, Islamic Azad University, Quchan, Iran; ^5^Department of Nutrition, Faculty of Medicine, Mashhad University of Medical Sciences, Mashhad, Iran; ^6^Applied Biomedical Research Center, Mashhad University of Medical Sciences, Mashhad, Iran; ^7^Biotechnology Research Center, Pharmaceutical Technology Institute, Mashhad University of Medical Sciences, Mashhad, Iran; ^8^Department of Biotechnology, School of Pharmacy, Mashhad University of Medical Sciences, Mashhad, Iran

## Abstract

Pathophysiological pathways that are induced by chronic hyperglycemia negatively impact lipid metabolism. Thus, diabetes is commonly accompanied by varying degrees of dyslipidemia which is itself a major risk factor for further macro- and microvascular diabetes complications such as atherosclerosis and nephropathy. Therefore, normalizing lipid metabolism is an attractive goal for therapy in patients with diabetes. Incretin-based medications are a novel group of antidiabetic agents with potent hypoglycemic effects. While the impact of incretins on glucose metabolism is clear, recent evidence indicates their positive modulatory roles on various aspects of lipid metabolism. Therefore, incretins may offer additional beneficial effects beyond that of glucose normalization. In the current review, how these antidiabetic medications can regulate lipid homeostasis and the possible cellular pathways involved are discussed, incorporating related clinical evidence about incretin effects on lipid homeostasis.

## 1. Introduction

Diabetes mellitus (DM) is an increasingly prevalent metabolic disorder, the major sign and symptom being hyperglycemia and polyuria, respectively [1]. This chronic disease is a major underlying cause for severe debilitating conditions such as cardiovascular disorders and renal failure [2]. Aberrations in the normal physiological metabolic pathways of most substrates, including lipids, are present in DM, and the disordered metabolism can induce the onset and progression of metabolic disorders [3–5]. Many diabetic complications involve dyslipidemia, and lipid homeostasis has obvious impacts on the function of most organs, important examples being the kidneys, heart, blood vessels, the neuronal network, and testes [6]. Therefore, normalizing lipid metabolism in the diabetic milieu is an important goal for prevention of diabetes-induced complications [3, 6].

Incretin-based medications are a new class of antidiabetic drugs that effectively lower circulating glucose, acting through various cellular pathways [7]. These antihyperglycemic agents have significant effects on body metabolism and increase insulin sensitivity via multiple molecular mechanisms [7]. Although some evidence indicates beneficial effects of these drugs on adipocytes and lipids [8], their exact role in lipid homeostasis is, to date, poorly understood. Should incretins be able to normalize lipid homeostasis, this would extend their therapeutic effects beyond their hypoglycemic role in diabetic patients. Therefore, in the current study, we present an update on current knowledge regarding the impact of incretins on lipid homeostasis.

## 2. Incretin-Based Antidiabetic Drugs

**Figure 1:**
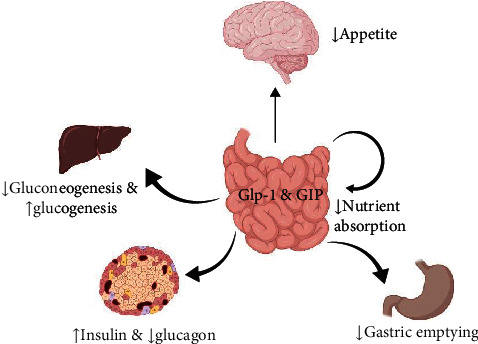
The major metabolic effects of incretins.

Incretins are a family of intestinal hormones that includes glucagon-like peptide-1 (GLP-1) and gastric inhibitory peptide (GIP) and that exerts their antidiabetic effects via diverse mechanisms such as glucagon release inhibition, stimulating insulin secretion, causing delay in gastric emptying and appetite suppression, reducing the absorption of intestinal nutrients, improving lipid metabolism, and promoting pancreatic *β*-cell function (Figure 1) [9–13]. GLP-1 is mainly secreted by intestinal enteroendocrine L-cells (as well as certain neurons within the nucleus of the solitary tract), while GIP is synthesized in the gastrointestinal tract by K cells located in the mucosa of the duodenum and jejunum [9–13]. The GLP-1 peptide acts by binding to its specific receptor, the GLP-1 receptor (GLP-1R), that is mainly located on pancreatic *β*-cells [11, 14]. GLP-1 binding to GLP-1R is followed by an increase in production of cyclic adenosine monophosphate (cAMP), cellular depolarization, and intracellular calcium augmentation, leading to insulin secretion from pancreatic *β*-cells [11, 14].

Two main classes of incretin antidiabetic drugs have been developed, the GLP-1 receptor agonists (GLP-1RAs) and dipeptidyl peptidase-4 inhibitors (DPP-4is) (Table 1) [9, 15]. Besides antidiabetic actions, incretin-based drugs have a plethora of beneficial effects on body organs [16–23]. GLP-1RAs reduce blood glucose by stimulating glucose-dependent insulin release from the pancreatic islets, while DPP-4i increase the circulatory level of endogenous GLP-1 by inhibiting the protease enzyme DPP-4, a serine exopeptidase which is physiologically responsible for GLP-1 metabolism and inactivation [9, 15]. DPP-4 inhibitors and GLP-1RA have similar hypoglycemic effects, although they may have some differences in pharmacological effects such as influence on body weight and risk of adverse effects [24] (Table 1).

## 3. Lipids, Physiology, and Metabolism

Lipids are hydrocarbonated micromolecules that are not soluble in water but can be dissolved in nonpolar solvents [26]. Due to differences in structure and function, there are numerous types of lipids: triglycerides (TG), phospholipids (PL), cholesterol (CLS), and lipidemic molecules such as sphingolipids, glycolipids, and prostaglandins [27]. TGs and the PLs consist of free fatty acids (FFA) (simple long-chain hydrocarbon organic acids with the common formula C_N_H_N_COOH), whilst CLS has no complete fatty acid in its structure [27]. However, CLS has many of the biochemical properties of the lipids since its nucleus is made of fatty acid-like biomolecules [27]. Lipids have many physiological functions, including energy storage, signaling activities, and structural functions [27]. Lipids are incorporated into the eukaryotic cell membrane structure by forming a double layer membrane known as a lipid bilayer [27]. They are also involved in the structure of many steroids and steroid hormones such as vitamin D_3_, prostaglandins, sex hormones, and adrenal steroids (glucocorticoids and mineralocorticoids) [27]. Therefore, lipids and their derivatives are closely involved in homeostasis and are vital for human health.

Adipose tissue (AT), a metabolic organ involved in energy homeostasis in the body, consists of fat that is largely composed of TGs and PLs [26, 28]. Originally considered to be inert tissue formed from storage of excess energy, AT was later shown to have important biological function, synthesizing biomolecules such as adipokines and adiponectins and releasing them into the circulation [28]. These peptides have significant hormonal effects on most metabolic pathways, and hence, AT is now recognized as an endocrine organ with significant metabolic impacts [6].

Lipid metabolism encompasses the processes of absorption, synthesis, polymerization, conversion, and degradation of the lipid molecules [26]. These processes are finely tuned, exhibiting a delicate dynamic equilibrium (in the healthy physiologic state) which ultimately determines the total fat mass of the body [26]. While some types of lipids are constantly being oxidized to provide for the metabolic needs for the body, others are being synthesized, replaced, and stored [26, 29]. Lipid metabolism is under the influence of many endogenous and exogenous factors [29] and is regulated by hormones such as growth hormone, sex steroids, adipokines, adrenal steroids, and thyroid hormones as well as neuronal stimuli [30]. Many physiological, pathological, and social stimuli, such as exercise, the intensity of physical activity, feeding habits, and stressors, are able to modulate lipid metabolism [30].

## 4. The Importance of Lipid Homeostasis in Health and Disease

Lipid homeostasis is critical for the function of most physiologic systems, examples being the cardiovascular system, kidneys, retina, and nervous system, and is therefore important for overall physical health [31]. A strong body of evidence links dyslipidemia to many life-threatening disorders such as atherosclerosis, nephropathy, fatty liver disease, and thrombosis [31, 32]. For example, hypercholesterolemia is an underlying cause of atheroma plaques, atherosclerosis, and myocardial infarctions [33]. Dyslipidemia also negatively impacts renal sufficiency [34] and retinal functions [35, 36]. In the diabetic state, conditions are more conducive for dyslipidemia and dysregulated fat metabolism [32], and it is likely that all diabetic complications are in some ways associated with dyslipidemia [37, 38]. Dyslipidemia can precipitate and progress diabetic complications via several pathologic pathways [38]. Therefore, the majority of patients with diabetes are prescribed lipid-modulating drugs in addition to antidiabetic agents to improve lipid homeostasis and help prevent the onset of diabetic complications [39].

## 5. Incretins and Lipid Homeostasis

Beyond their hypoglycemic effects, incretin-based drugs exert modulatory effects on various steps of lipid metabolism [40, 41] (Table 2). These effects can help diabetic patients to normalize fat metabolism whilst concurrently normalizing glycemic levels [39]. In the following sections, we will discuss these metabolic effects.

### 5.1. Lipogenesis and Lipolysis

Lipolysis and lipogenesis are the two main metabolic processes in lipid metabolism [42]. They are key determinants of adiposity, and the amount of stored lipids is achieved by promoting or reducing lipid reserves in adipocytes [43]. Lipogenesis is the metabolic process in which FFA and TG are synthesized from different substrates, such as carbohydrate, acetyl-coenzyme A (CoA), and glycerol [44]. FFAs are synthesized in the cytoplasm of the cells (specifically the mitochondria) from acetyl-CoA (in a process known as the *de novo* pathway), whilst TG synthesis takes place mainly in the membrane of the smooth endoplasmic reticulum (SER) [44]. Both processes occur primarily in the liver and adipose tissue, but in individuals with a high caloric diet and where there is high carbohydrate availability, adipose tissue functions as the major site of lipogenesis [44]. However, other tissues, such as the kidneys, brain, lung, and gut, may also produce lipids to some extent [45]. Lipogenesis is a highly controlled metabolic process [46, 47] and is influenced by both stimulatory and inhibitory factors such as transcriptional elements, hormones, and metabolic enzymes [47]. Uncontrolled or pathological lipogenesis is directly linked to metabolic disorders and obesity, and therefore, many lipogenesis-modulating pharmacologic agents have been developed [48].

Lipolysis (or the degradation of lipid molecules into their constituent parts) is the other key lipid metabolic process which uniquely occurs in white adipocyte tissue [43]. In this process, TGs break down into their constituent molecules, as FFAs and glycerol, via hydrolysis [43]. Lipolysis takes place mainly at the surface of cytosolic lipid droplets in adipocytes and releases the FFAs and glycerol for use by other tissues [43]. During fasting, lipolysis is induced in order to supply the required FFAs for oxidative metabolism [49]. Lipolysis also prevents serum FFA increase which may result in lipotoxicity [50]. As with lipogenesis, the lipolytic process is under the influence of many stimuli and imbalances impacting lipid metabolism may induce a wide array of metabolic disorders such as obesity, insulin resistance, atherosclerosis, nonalcoholic fatty liver disease (NAFLD) and DM [51]. Therefore, the appropriate lipolytic balance is of great importance in diabetes to prevent downstream complications [51].

Incretin-based drugs are able to modulate both lipogenesis and lipolysis [42]. Sancho et al., in an *in vivo* study, found that GLP-1 impacts kinases, such as PI3K, p44, and p42 MAPKs and possibly PKC, that are involved in lipolytic and lipogenic processes [42]. They found that GLP-1 and its agonists greatly impact lipogenesis and lipolysis in rat adipocytes [42]. Recent evidence confirms that GLP-1 interacts with the major lipid metabolic enzymes such as lipase [52, 53], pyruvate dehydrogenase [54], acetyl-CoA carboxylase [55, 56], and fatty acid synthase [57].

Ben-Shlomo and colleagues in 2011 demonstrated that GLP-1 inhibits lipogenesis via an AMPK-dependent pathway in high fat diet (HFD) rats [58]. They found that GLP-1 therapy suppressed lipogenic enzymes such as sterol response element binding protein-1c (SREBP-1c), stearoyl CoA desaturase-1 (SCD-1), fatty acid synthase (FAS), and carnitine palmitoyl transferase-1 (CPT-1) in hepatic cells of experimental rats [58]. Parlevliet et al. in 2012 provided further evidence demonstrating that both GLP-1RA (CNTO3649) and DPP-4i (exendin-4) decrease hepatic lipogenesis in HFD mice [59]. They found that GLP-1 therapy downregulates the genes involved in lipogenesis such as SREBP-1c, FAS, diacylglycerol O-acyltransferase 1 (Dgat1), and apolipoprotein B synthesis (ApoB) [59]. Moreover, Ideta and coworkers in 2015 reported that the DPP-4i teneligliptin reduces hepatic lipogenesis by an AMPK-dependent process in mice [60]. They showed that teneligliptin therapy activates the AMPK pathway and attenuates expression of lipogenic genes to improve NAFLD in a mouse model [60]. A signaling axis between GLP-1 and lipogenesis was suggested by more studies in which incretin-based medications ameliorate lipogenesis via AMPK activation pathway [57, 61]. GLP-1 therapy may also increase postprandial chylomicron synthesis, thereby increasing fat storage and normalizing serum lipid levels [62]. The weight of evidence therefore suggests that GLP-1 therapy is promising for modulation of lipogenesis in diabetic patients [63].

Incretin-based medications may also be able to modulate lipolysis [64]. GLP-1 agonists reduce adiposity by induction of lipolysis [65]. The first evidence for this was reported by Ruiz-Grande et al. in 1992 [66]. They found that GLP-1 [1–36] exerts lipolytic effects on cultured adipocytes of rats via stimulating different receptors than for the glucagon hormone [66]. Yaney et al. in 2001 demonstrated that GLP-1 induces lipolysis and releases FFAs in a cAMP-dependent manner in clonal pancreatic beta cells [67]. In another study, GLP-1 was able to induce lipolysis only at high doses in human isolated adipocytes [68]. In this study, GLP-1 exerted dual effects on lipid metabolism via inhibition of lipolysis and promotion of lipogenic pathways at a low concentration [68]. Xu and colleagues in 2016 provided further evidence demonstrating that exendin-4 promoted lipolysis via potentiation of antioxidative defenses in 3T3-L1 adipocytes [49]. They found that exendin-4 increases phosphorylated hormone-sensitive lipase (HSL), a major hallmark of lipolysis [49]. Other evidence by Patel et al. in 2017 established that a synthesized agonist of GLP-1R (known as Aib2 C24 chimera) controlled lipolysis and modulated dyslipidemia in obese hamsters [69]. Recent clinical evidence from 2019 reported that liraglutide, a GLP-1RA, reduced adiposity and body fat mass by promoting lipolysis in obese patients with type 2 diabetes mellitus (T2DM) [70]. More recent evidence presented by Pereira et al. indicated that GLP-1R induction increases lipolysis and reduces adiposity in human adipocytes [71]. A study by Rago and coworkers in 2020 reported that human sperm possess GLP-1R that enables them to control lipid metabolism via lipogenic and lipolytic pathways [72], a finding that emphasizes the role of these receptors in lipid homeostasis [72]. Thus, incretin-based therapies could be promising agents for improving dyslipidemia and lipid homeostasis [73] by altering the balance between lipogenesis and lipolysis toward lower adiposity and prevention of diabetes-related dyslipidemia-induced complications such as diabetic nephropathy [74, 75] and beta cell dysfunction [76].

### 5.2. Lipid-Peroxidation/Lipotoxicity

Lipid peroxidation is an injurious cellular event caused by oxidative degradation of lipids in which free radical species procure electrons from cell membrane lipids and, in turn, produce toxic byproducts such as lipid hydroxides [77]. Different forms of these byproducts, such as malondialdehyde (MDA), F2-isoprostanes, and 4-hydroxynonenal (HNE), are increased in oxidative stress and are therefore recognized as biomarkers of oxidative damage [77]. These byproducts are also able to bind to DNA at specific points and promote mutations by forming DNA adducts, thereby producing additional biomarkers such as 8-oxo-2′-deoxyguanosine (8oxodG) [78]. These events most commonly occur in environments with weakened antioxidant capacity such as diabetes [79].

GLP-1 mimetics have varying abilities to protect against lipid peroxidation [80]. These drugs have potent direct and indirect antioxidative capacities (such as antioxidant defense system (ADS) potentiation, prooxidant inhibition, steroid receptor coactivator (SRC) protein suppression, and improvement in mitochondrial function) that enable them to prevent oxidative stress-induced lipid peroxidation [21]. Patel and colleagues in 2013 reported that GLP-1 therapy prevented lipid peroxidation via improving oxidative stress in mice [80]. Another study reported that exenatide reduced MDA content (a marker of lipid peroxidation) in patients with T2DM [81]. A recent article confirmed these reports and demonstrated that the GLP-1 mimetic, myricetin (a novel DPP-4i), reduced lipid peroxidation in the oxidative milieu of diabetic mice [82]. Therefore, clear evidence indicates that incretin-based therapies inhibit lipid peroxidation via increasing ADS potency and reducing oxidative stress.

**Figure 2:**
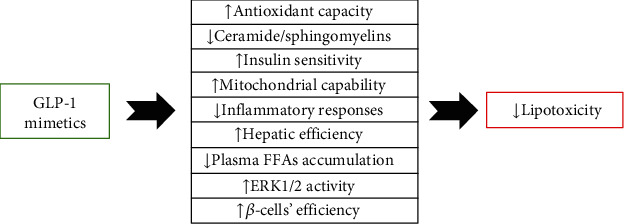
The antilipotoxicity effects of GLP-1 mimetics.

GLP-1 mimetics have also been suggested as key therapeutic agents for prevention of lipotoxicity [83], functioning through several pathways (Figure 2). These drugs combat excess serum lipid levels through their potent anti-inflammatory and antioxidative actions [83]. Huang and coworkers in 2015 demonstrated that liraglutide mitigates inflammation and oxidative stress and promotes beta cell proliferation and improved islet function [83]. Armstrong et al. in 2016 reported that liraglutide inhibits lipotoxicity in patients with NAFLD by increasing insulin sensitivity in adipocytes and improving liver function [84]. Gu et al. in 2016 demonstrated that exendin-4 attenuates lipotoxicity through ERK1/2 activation and improvements in mitochondrial function [85]. Another proposed mechanism of action, as reported by Somm et al. in 2021in mice, is through prevention of ceramide accumulation [86]. Liu et al. in 2020 reported that GLP-1 therapy reduces lipotoxicity by suppression of the inflammatory mediator NF-*κ*b in mice [87].

It has also been suggested that impaired GLP-1 secretion is associated with dyslipidemia (hypertriglyceridemia) and the degree of lipotoxicity [88]. Wang and colleagues in 2018 found that diabetic patients with dyslipidemia have impaired GLP-1 secretion that directly correlated with lipotoxicity [88]. Moreover, GLP-1 secretion may be increased in response to lipotoxicity and dyslipidemia in an effort to mitigate them [89]. In summary, incretin-based medications have pharmacological properties that enable them to inhibit lipotoxicity and thereby help to prevent lipotoxicity-dependent disorders, especially renal and cardiovascular complications [90].

### 5.3. Fatty Acid *β*-Oxidation

Fatty acid *β*-oxidation is a polyphasic enzymatic process in which FAs (long-chain acyl-CoA) are broken down to acetyl-CoA to produce energy [91]. This catabolic process occurs in mitochondria (as well as peroxisomes) to generate acetyl-CoA followed by nicotinamide adenine dinucleotide (NADH) and flavin adenine dinucleotide 2 (FADH2) which, in turn, enter the citric acid cycle and the mitochondrial electron transport chain, producing energy as adenosine triphosphate (ATP) [91]. This process consumes the FAs, thereby lowering serum FA levels and the overall body fat supply; it therefore plays a significant role in lipid homeostasis and determining the amount of fat in body tissues [91] as well as maintaining tissue energy balance [92]. Fatty acid *β*-oxidation is regulated at two major levels: transcriptional and posttranscriptional or allosteric [91]. While transcriptional regulation is performed by key proteins such as peroxisome proliferator-activated receptors (PPARs), sterol regulatory element-binding protein 1 (SREBP1), and peroxisome proliferator-activated receptor-*γ* coactivator-1*α* (PGC-1*α*), allosteric control is carried out by the level of by-products produced which may negatively or positively affect the relevant metabolic enzymes [91]. Some evidence indicates that incretin-based medications can interact with these regulators [49, 93].

Xu and colleagues in 2016 demonstrated that exendin-4 induces fatty acid oxidation via a sirtuin 1- (SIRT-1-) dependent signaling pathway in 3T3L1 adipocytes [49]. They found that GLP-1 signaling improves oxidant capacity which, in turn, increases FA oxidation in cultured adipocytes [49]. A clinical study in 2019 demonstrated that liraglutide induces lipid oxidation and reduces adiposity in obese patients with T2DM [70]. Timper et al. in 2020 found that GLP-1R signaling promotes FA *β*-oxidation in cultured astrocytes which, in turn, improves lipid homeostasis and memory efficiency, suggesting that GLP-1 signaling is important to energy homeostasis and brain function through FA *β*-oxidation-dependent pathways [94]. Further studies have suggested that GLP-1 signaling exerts protective effects on energy homeostasis by promoting fatty acid oxidation [92]. GLP-1 can improve insulin sensitivity in hepatocytes by promoting hepatic fatty acid oxidation in both human and rat liver biopsies [95]. GLP-1 signaling induces an intrinsic signal to increase fatty acid oxidation and reduce insulin resistance in the diabetic state [96]. GLP-1 also interacts with genes involved in FA *β*-oxidation [93]. Recent evidence suggests that GLP-1 receptors expressed on cardiomyocytes provide cardioprotection through interactions that increase fatty acid beta-oxidation that, in turn, reduce epicardial adipose tissue thickness and improve cardiac function [93]. Taken together, this evidence demonstrates that GLP-1 signaling has a significant impact on fatty acid oxidation and may provide some of its beneficial effects as an insulin sensitizer and cardioprotective agent through modulation of FA *β*-oxidation.

### 5.4. Cholesterol Synthesis

Cholesterol is a lipid molecule which is synthesized by most eukaryotes [97]. It is involved in many important biologic activities, such as hormone synthesis and cell membrane formation [97]. However, in higher concentrations, it becomes a risk factor for cardiovascular disease [75]. Therefore, maintaining cholesterol in the physiologic range is critically important [75]. Recent evidence suggests that GLP-1 mimetics have a modulatory role on cholesterol homeostasis and may improve impaired cholesterol metabolism [98, 99].

Yao et al. in 2018 found that GLP-1 affects both cholesterol transport and synthesis, modulating cholesterol homeostasis via upregulation of ABCA-1and downregulation of miR-19b in isolated hepatocytes [98]. Kim et al. in 2020 demonstrated that GLP-1 improves brain function via improvement in cholesterol metabolism, suggesting that GLP-1's ability to regulate cerebral blood flow is dependent upon cholesterol homeostasis [99]. A study in 2018 demonstrated that the GLP-1 analogue liraglutide reduced circulating cholesterol levels in mice on a high fat diet [100]. Similar evidence from 2018 showed that GLP-1 receptor agonists reduce cholesterol synthesis by suppressing the HMG-CoA reductase (a key enzyme in cholesterol synthesis) and SREBP-1C [101]. These transcriptional effects of GLP-1 are cardioprotective by reducing atheroma plaque formation [101]. Clinical evidence from 2018 demonstrated that GLP-1 administration reduces serum cholesterol levels and improves dyslipidemia by inhibition of HMG-CoA reductase in T2DM patients [102]. These findings strongly suggest that GLP-1 mimetics have a positive modulatory impact on cholesterol metabolism and, in turn, dyslipidemia and atheroma plaque formation and therefore provide further protective lipid-modulating effects beyond their antidiabetic effects.

### 5.5. Lipid Absorption

Incretins are key regulators of intestinal lipid absorption [8]. GLP-1 has regulatory effects on satiety through the melanocortin 4 receptor- (MC4R-) mediated sympathetic system and by reducing lipid storage in both hepatic and adipose tissues [103]. Since GLP-1 is produced and secreted by intestinal L-cells and acts directly on the small intestine, it was suggested as likely that it affected intestinal lipid absorption [103]. Further studies showed that GLP-1 reduces postprandial chylomicron and TG circulatory levels by reducing intestinal lipid absorption [104]. GLP-1R agonists, such as exendin-4 (but not GIP), reduced intestinal lipid absorption and circulating levels of both triacylglycerol and ApoB-48 in an animal model on high fat diet [105]. GLP-1 also reduced intestinal lymph flow and TG absorption [106]. These findings suggest that endogenous GLP-1 regulates postprandial lipaemia through several pathways in addition to controlling lipid absorption [105].

## 6. Conclusion

**Figure 3:**
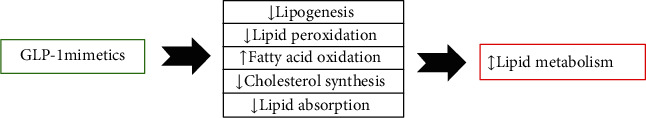
The different impacts of incretin-based medications on lipid metabolism.

Incretin-based medications are a novel class of antidiabetic drugs that provide potent modulatory effects on glucose metabolism. However, much evidence suggests that they also regulate lipid metabolism. Since most diabetic patients have some degree of dyslipidemia, the lipid-modulatory effects of incretins provide further pharmacological benefits for diabetic patients. Our review demonstrates that incretin-based therapies modulate lipid metabolism via at least five cellular pathways: lipogenesis and lipolysis, lipid peroxidation, lipid absorption, cholesterol biosynthesis, and fatty acid beta-oxidation (Figure 3). We also present clinical evidence supporting the experimental findings. However, more experimental and clinical studies are still needed to further elucidate the molecular targets that are affected by incretin-based therapeutics to modulate lipid and lipoprotein metabolism. Moreover, evidence from lipoprotein tracer kinetic studies will pave the way towards elucidation of the impact of incretin-based agents on lipoprotein biogenesis and catabolism. There is also a lack of enough clinical evidence about the effects of incretin-based therapies on the risk of diabetic macrovascular and microvascular complications. Finally, the possible role of combining incretin-based drugs with common lipid-lowering agents in nondiabetic individuals remains open to question.

Taken together, this body of evidence suggests that incretin-based medications are effective antidiabetic therapies especially in patients with dyslipidemia, as they have the potential to normalize lipid metabolism in diabetes.

## Figures and Tables

**Table 1:** The two main classes of incretin-based antidiabetic drugs.

Classes	Approved forms	Mechanisms of action	Ref.
GLP-1RA	Exenatide (exendin-4), Albiglutide, liraglutide, lixisenatide, semaglutide, dulaglutide	Agonists of intrinsic incretins	[9, 15]
DPP-4i	Sitagliptin, saxagliptin, vildagliptin, linagliptin	Inhibit incretin inactivation	[24, 25]

**Table 2:** The impact of incretin-based medications on lipid metabolism.

Lipid metabolism	Effects of incretin-based therapy	Ref.	Clinical evidence
Lipogenesis and lipolysis	Reduces lipogenesis mainly thru an AMPK-dependent pathway	[57–61, 63, 74]	[63, 70, 107, 108]
Lipid peroxidation	Inhibits lipid peroxidation and reduces lipotoxicity	[21, 80–82, 84]	[88]
Fatty acid oxidation	Induces and promotes fatty acid oxidation	[49, 95]	[93]
Cholesterol synthesis	Inhibits HMG-CoA reductase and cholesterol biosynthesis, reduces atheroma plaque formation	[98, 100, 101]	[102]
Lipid absorption	Commonly reduces intestinal lipid absorption	[103, 104]	[105]

## Data Availability

There is no raw data associated with this review.
